# Incorporation of a β-AgVO_3_ Semiconductor in Resin Cement: Evaluation of Mechanical Properties and Antibacterial Efficacy

**DOI:** 10.3290/j.jad.b2916423

**Published:** 2022-04-13

**Authors:** Simone Kreve, André Luís Botelho, Mariana Lima da Costa Valente, Luciano Bachmann, Marco Antônio Schiavon, Andréa C. Dos Reis

**Affiliations:** a Doctoral Student, Department of Dental Materials and Prosthodontics, Ribeirão Preto School of Dentistry, University of São Paulo, Ribeirão Preto, SP, Brazil. Primarily responsible for research methodology, drafted the manuscript.; b Postdoctoral Student, Department of Dental Materials and Prosthodontics, Ribeirão Preto School of Dentistry, University of São Paulo, Ribeirão Preto, SP, Brazil. contributed significantly to the study design and execution of the research methodology.; c Postdoctoral Student, Department of Dental Materials and Prosthodontics, Ribeirão Preto School of Dentistry, University of São Paulo, Ribeirão Preto, SP, Brazil. Statistical analysis, data interpretation.; d Professor, Department of Physics, University of São Paulo, School of Philosophy, Sciences and Letters of Ribeirão Preto, Ribeirão Preto, São Paulo, Brazil. Performed FTIR experiments, drafted the manuscript.; e Professor, Department of Natural Sciences, Federal University of São João del-Rei (UFSJ), São João del-Rei, Brazil. Synthesized nanostructured silver vanadate, drafted the manuscript.; f Professor and Coordinator of the Postgraduate Program, Department of Dental Materials and Prosthodontics, Ribeirão Preto School of Dentistry, University of São Paulo, Ribeirão Preto, SP, Brazil. Project coordinator, research supervisor, contributed to data interpretation, reviewed the manuscript.

**Keywords:** mechanical properties, surface roughness, resin cements, silver vanadate, nanomaterials

## Abstract

**Purpose:**

This in vitro study aimed to investigate the effect of incorporating the semiconductor nanostructured silver vanadate decorated with silver nanoparticles (β-AgVO_3_) in a dual-cure resin cement on the degree of conversion (DC), microhardness, roughness, color, adhesion properties before and after artificial aging, and antimicrobial efficacy.

**Material and Methods:**

Three test groups were established: control (without β-AgVO_3_), with the incorporation of 2.5% and 5% (by weight) of β-AgVO_3_ in dual-cure resin cement (Allcem, FGM). The degree of conversion was measured using Fourier transform infrared spectroscopy (FTIR). To evaluate roughness (n = 10), microhardness (n = 10), color (n = 10), and to perform agar disk diffusion (n = 8), disks of 6-mm diameter and 2-mm height were manufactured using the same concentrations. For the color and shear bond strength test (n = 6), orthodontic brackets (Morelli) were used, which were cemented to natural human enamel and evaluated before and after artificial aging via thermocycling at 5°C and 55°C for 1000 cycles. For color measurements, a portable spectrocolorimeter and the CIE-Lab method were used. Data were analyzed using Student’s t-test, ANOVA, and Tukey’s multiple comparisons with significance set at α = 0.05.

**Results:**

Semiconductor incorporation did not influence the cements’s DC. The incorporation of 2.5% and 5% of β-AgVO_3_ resulted in a significant increase in Knoop microhardness and surface roughness. Significant changes were observed in the color of the specimens when the semiconductor was incorporated. Adhesion after aging remained within the clinically recommended values in all groups, and antimicrobial activity was observed against *Staphylococcus aureus, Streptococcus mutans,* and *Enterococcus faecalis* at both concentrations tested.

**Conclusion:**

It is suggested to incorporate the semiconductor β-AgVO_3_ in the dual-cure resin cement at both concentrations. Moreover, the physical-mechanical properties remained satisfactory for the proposed application.

Semiconductors are materials with technological importance for the electronics industry and environmental remediation due to their ability to generate charge carriers when activated with a certain energy. For example, semiconductors have excellent electron-conducting ability and responsiveness to light in the UV-visible region.^14,23^ These materials are widely used for sensing management, photocatalytic energy generation (photocatalysis), and electrochemical applications.^29^ Regarding photocatalysis, research to date has been focused for instance on pollutant degradation,^29^ water treatment,^57^ and solar power generation.^23,63^

More recently, metal deposition on semiconductors for use as photocatalysts has shown promise,^12,23^ attracting interest from areas such as dentistry due to their possible applications as potential antibacterial agents. In particular, it was observed that metal nanoparticles incorporated into the semiconductor matrix result in excellent antibacterial and catalytic properties.^12,17,63^ Photocatalyst semiconductors kill bacteria by direct contact, promote cell membrane disruption, generate reactive oxygen species (ROS), penetrate through the bacterial membrane, etc.^12,17,63^

One example of a semiconductor being studied in dentistry^13,15,39,59,60^ is based on silver vanadium oxide.^17^ Nanostructured silver vanadate decorated with silver nanoparticles (β-AgVO_3_)^17,32^ is synthesized by a precipitation reaction of ammonium vanadate (NH_4_VO_3_) and silver nitrate (AgNO_3_), followed by hydrothermal treatment.^32^ This material has the advantage of being stable, and its properties, such as size, shape, crystal structure, can be adjusted. This semiconductor has a large surface area, which ensures a higher antimicrobial potential.^32^ In addition, this material has the important characteristic of not agglomerating, unlike other silver nanoparticle-based materials that usually do.^22,46^ Such agglomerations act in a manner similar to that of impurities, performing as centers of stress concentration in the matrix, reducing the mechanical properties.^16^

In dentistry, advances in esthetic adhesive procedures have intensified the use of resin cements. However, problems associated with secondary caries at the tooth/restoration interface, the presence of white enamel lesions around orthodontic brackets, marginal deterioration of cemented restorations, and discoloration at the interface highlight the need for the development of adhesive resin materials with antibacterial properties.

Studies with this aim were conducted using antimicrobials such as chlorhexidine^30^ and benzalkonium chloride,^1^ but the results showed antimicrobial leaching,^30^ lack of antimicrobial effectiveness against *Enterococcus faecalis*,^30^ and changes in mechanical properties.^1^ Given this background, interest in combining the advantages of semiconductors with the need to introduce antimicrobial activity into resin cements has arisen.

The semiconductor β-AgVO_3_ has already been incorporated into acrylic resins,^13^ soft liners,^39^ irreversible hydrocolloid impressions,^14^ and endodontic cements,^59^ showing promising results. But there is still a gap regarding resin cements.

This study proposed to evaluate the degree of conversion (DC), color, microhardness, surface roughness, and adhesion using orthodontic brackets, because this would enable us to observe a group of properties that can be used in other dental applications.

It is known that incomplete conversion of monomers into polymeric chains and the presence of unconverted free monomers (ie, reduced DC)^45^ reduced hardness and compressive strength, and increases solubility^18^ and oral fluid absorption.^54^ In the oral cavity, restorations are subjected to mechanical, chemical, and thermal stress and thus undergo a process of aging.^10^ This can alter the color and negatively influence the adhesive properties.^28^ In this respect, thermocycling is the most commonly used test to simulate the effect of temperature changes in the oral cavity.^10^

Two hypotheses were tested: 1) the incorporation of β-AgVO_3_ into resin cement maintains the original properties of the cement unchanged; and 2) the incorporation of β-AgVO_3_ provides an antimicrobial effect against *Staphylococcus aureus, Streptococcus mutans,* and *Enterococcus faecalis*.

## Materials and Methods

### Synthesis of Nanostructured Silver Vanadate Decorated with AgNPs

This material was synthesized as previously described.^32,33^ The reaction occurred with the precipitation of silver nitrate (Merck 99.8%) and ammonium metavanadate (NH_4_VO_3_, Merck 99%). Initially, 1 mmol NH_4_VO_3_ was solubilized in 35 ml of distilled water at 80°C, under magnetic stirring for 15 min. Then, 2 mmol of AgNO_3_ were added dropwise to 35 ml of distilled water, then stirred for 30 min to form the silver vanadate solution. The precipitate obtained was centrifuged, washed with distilled water and absolute ethanol, and dried under vacuum conditions for 10 h.

### Preparation of Specimens for Microhardness, Roughness, and Microbiological Testing

This study used Allcem dual-cure resin cement (FGM; Joinville, Santa Catarina, Brazil). Table 1 shows the chemical composition of the material. A total of 72 specimens were produced, and of these, 30 (n = 10) were used in the surface roughness and microhardness tests. After these tests, all 72 specimens were sterilized with hydrogen peroxide gas plasma^46^ for the halo test (n = 8). A cylindrical Teflon matrix 6 mm in diameter and 2 mm in height was used to produce the specimens.^25^ Three study groups were formed: 1) control (without adding β-AgVO_3_); 2) addition of 2.5% β-AgVO_3_ (wt%); 3) addition of 5% β-AgVO_3_ (wt%). The cement preparation followed the manufacturer’s instructions. For the groups containing β-AgVO_3_, the cement was placed on a glass plate and weighed on a digital scale. Thus, the amount of β-AgVO_3_ powder was proportioned and added to the cement. After mixing, the mixture was inserted into the Teflon matrix and light cured with an Optilux 501 curing unit (Kerr; Orange, CA, USA) for 40 s at 800 mW/cm^2^ of energy. After fabrication, the specimens were wet ground with 600-, 800-, and 1200-grit sandpaper. At the end of this process, the specimens were measured using a digital pachymeter (Mitutoyo; Kawasaki, Japan) to check the dimensions, and stored in a light-proof box for 24 h.^48^

**Table 1:** Composition of dual-curing resin cement

Resin cement	Composition
Allcem dual-cure	Base paste: bis-GMA, bis-EMA, TEG-DMA, camphoroquinone barium-aluminum-silicate microglass, sílica nanoparticles. Catalyst paste: methacrylic monomers, dibenzoyl peroxide and stabilizers, barium-aluminum-silicate microparticles of 66-67 wt% of the mixture.

**Table 2:** Progression of the DC% as a function of time

Time point	Control	2.5% group	5% group
1 min	70.5%	70.0%	65.3%
5 min	74.5%	69.8%	72.1%
10 min	76.3%	76.1%	74.4%
48 h	63.8%	69.2%	71.4%

The sample size for the microbiological assay (n = 8) was determined by a power test using R software. The data used included a standard deviation of 1.73%, effect magnitude of 2.33%, power of 0.8, and significance level of p = 0.05.^27^

### Degree of Conversion

The DC of the monomer (DC%) was assessed by Fourier-transform infrared spectroscopy (FT-IR) (Nicolet 380, Thermo Nicolet; Waltham, MA, USA) using a universal attenuated total reflection (ATR) sampling accessory. After mixing the base and catalyst pastes, the cement was placed in a stainless-steel ring (0.6 mm thick and 6.5 mm in diameter) located on the surface of the ZnSe-ATR crystal (1.6 mm penetration depth) and evaluated. The cement was then manually irradiated with a light-curing unit (Optilux-501, Kerr) for 40 s at 800 mW/cm^2^ of energy. The cement absorption spectrum was obtained at a resolution of 1 cm^-1^, and the absorption bands of the aromatic bonds (AbsC-C) and the aliphatic double bonds (AbsC = C) were recorded. The C=C band at 1638 cm^-1^ was divided by the area of the band C-C at 1605 cm^-1^. The measurements were performed in triplicate for each specimen before cement irradiation, then immediately, 5 min, 10 min, and 48 h after irradiation. The specimens were stored for 48 h in light-proof boxes. We calculated the percentage of remaining double bonds (RDB) using the following formula:

RDB% = (AbsC=C/AbsC-C for polymerized resin) x 100

(AbsC=C/AbsC-C for non-polymerized resin)

We calculated the conversion of double-bonds to single bonds in percent (DC%) based on FTIR using the following formula:

DC% = 100 – RDB%

### Surface Roughness

We evaluated the surface of the disks (n = 10) using a confocal laser microscope (Lext OLS 4000, Olympus; Tokyo, Japan) by acquiring successive sample images between valleys and peaks. Then, we recombined the acquired laser images, in color, to produce a 3D projection that can be measured. The 20X magnification objective was used to acquire the images, aiming at a qualitative analysis by visualizing the roughness characteristics. Quantitative data (average roughness, Sa) were also obtained by taking three measurements in different directions, then calculating the mean.

### Knoop Microhardness

In this test, the sample surfaces (n = 10) were printed with a diamond indenter at a 25-gF load (0.245 N) for 5 s.^15^ The microhardness value of each specimen consisted of the arithmetic mean of three random markings made on the surface of the specimens. After removing the load, we measured the notch diagonals with an optical microscope. The microhardness value was defined as the ratio between the indentation load and the residual impression area.

### Shear Bond Strength Test

This study was approved by the ethics committee of the Ribeirão Preto School of Dentistry, and received the CAAE number 20214019.5.0000.5419. The shear bond strength was determined after bonding the brackets and after aging. One-half of the samples (n = 18) were used to evaluate the shear bond strength immediately after bracket bonding, and the other half (n = 18) were aged prior to SBS testing.

#### Bracket bonding

Using a resin cement (Morelli; Sorocaba, São Paulo, Brazil), metal brackets were bonded to the enamel of natural, maxillary premolars acquired from the tooth Biobank of the Ribeirão Preto School of Dentistry (FORP/USP). For this, an “x” was marked on the buccal surface of the enamel and the bracket was positioned centered on this marking. The teeth used had been extracted for orthodontic or periodontal reasons. They were intact, without caries, cracks, or evident defects. A total of 36 teeth were used, n = 12 for each tested group:^34,53^ 1) control (without adding β-AgVO_3_); 2) addition of 2.5% β-AgVO_3_; 3) addition of 5% β-AgVO_3_ 37% phosphoric acid was applied for 30 s, then thoroughly rinsed, after which the adhesive (Ambar, FGM) was applied and light cured for 40 s. The cement-coated bracket was placed onto the tooth under light pressure, the excess cement was remove, and light curing was performed for 40 s. After bonding, the teeth bearing brackets were stored in distilled water for 24 h. The teeth were then positioned with the aid of a parallelometer into PVC tubes filled with acrylic resin so that only the roots of the teeth were submerged in the resin, leaving the crown with the bracket exposed. The PVC tubes were placed in a universal testing machine (EMIC DL 500, Emic Equipamentos e Sistemas de Ensaio; São José dos Pinhais, Paraná, Brazil) with a load cell positioned in the bracket channel. A 50-kgf force at a constant speed of 0.5 mm/min was applied until the bracket detached. Shear bond strength was recorded in MPa and performed in groups with and without artificial aging.

#### Artificial Aging

Artificial aging was performed using a thermocycling machine (MSCT-3; São Carlos, SP, Brazil) at 5°C and 55°C for 1000 cycles, with a complete cycle of 65 s (30-s dwell time, 5-s transfer time).^42^ For this test, the other half of the teeth (n = 18) were used, with n = 6 for each of the 3 groups tested.^34,53^

### Color Measurement

A portable spectrocolorimeter model Color Guide 45/0 (BYK-Gardner; Geretsried, Germany) was used to evaluate the color of the resin cement after the incorporation of the semiconductor (n = 10). This spectrocolorimeter uses the standard Commission Internationale de L’Eclairage (CIE Lab) color system, recommended by the American Dental Association. The preparation and finishing of specimens for this test followed the same protocols as for the microhardness, roughness, and microbiological analyses. The color was evaluated on the specimen’s surface. To eliminate the color difference from the background, a white pattern was placed on the specimen. The color change between specimens, given in terms of L, a, and b, was calculated using the following formula: ∆ Eab = [(∆L)^2^ + (∆a)^2^ + (∆b)2]^1⁄2^.

### Analysis of Antimicrobial Activity

The microorganisms *Staphylococcus aureus* (ATCC 6538), *Streptococcus mutans* (ATCC 25175), and *Enterococcus faecalis* (ATCC 29212), obtained from thawing the strains and cultivated at 37º C for 24 h in a microbiological oven, were centrifuged at 6000 g for 5 min. The supernatant was discarded and the pellet was washed twice with 10 ml PBS (phosphate buffered saline). The inoculum suspensions were made in BHI (brain heart infusion) medium, and the concentration was confirmed by optical density using a spectrophotometer (BEL Photonics, model 1105; Piracicaba, SP, Brazil), yielding absorbance readings of 0.150 for *E. faecalis*, 0.8 for *S. aureus*, and 0.108 for *S. mutans* at a wavelength of 625 nm, which corresponds to 10^8^ cells/ml. After standardization of the suspensions, the inocula were diluted to 10^6^ CFU ml^-1^. The BHI medium was used for *S. mutans*, and Mueller Hinton agar was used for *S. aureus*, and *E. faecalis*. The agar diffusion method was performed with n = 8 for each percentage of nanomaterial, and results were evaluated for the three microorganisms. The samples were placed in Petri dishes containing the culture media. The culture media for diffusion were heated at 50ºC, then the microorganisms were added in the established concentration. After being placed on the dishes, they rested for 2 h and were later stored for 24 h in an oven. Then, the zones of inhibition that formed around the specimens were measured using Image J software (National Institutes of Health; Bethesda, MD, USA). For each sample, the zone of inhibition was determined in mm in 3 regions, and the arithmetic mean was determined.

### Statistical Analysis

After checking the data distribution (Shapiro-Wilk test), ANOVA was used, followed by Tukey’s test, for Knoop microhardness, variables L*, a* and b* of the color measurement, shear bond strength, and antimicrobial activity. The Kruskal-Wallis test was used for surface roughness; Student’s t-test was used to evaluate ΔE*. Statistical significance was set at p < 0.05. Statistical analysis was performed with SPSS v 20.0 (Armonk, NY, USA).

## Results

### Degree of Conversion

**Fig 1:**
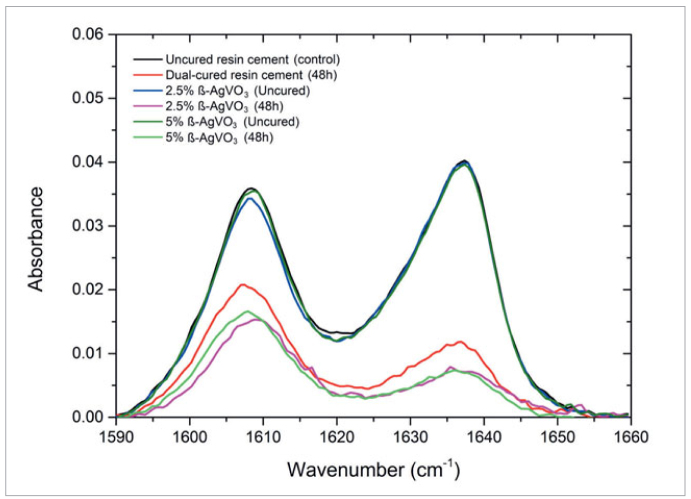
FTIR spectra of materials at different time points.

The spectra of the cement samples were recorded. A small amount of uncured resin cement was scanned and its spectrum was determined as the control group. The data obtained for the DC% are shown in Table 3. The incorporation of β-AgVO_3_ had no influence on the degree of conversion (Fig 1).

**Table 3:** Knoop microhardness (HK) and surface roughness (μm) of resin cement specimens incorporated with different percentages of nanostructured silver vanadate (β-AgVO_3_)

	Groups	Mean (standard deviation)	Median [confidence interval]
Knoop microhardness (HK)	Control	46.0 (3.2)A	45.1 [43.67; 48.30]
	2.5%	51.3 (1.5)B	51.30 [50.20; 52.33]
	5%	51.6 (1.4)B	51.75 [50.57;52.64]
Surface roughness (μm)	Control	0.2 (0.1)	0.16 [0.12;0.30]A
	2.5%	0.5 (0.4)	0.39 [0.24;0.81]B
	5%	0.6 (0.6)	0.40 [0.18;0.99]B

Identical letters indicate statistical similarity between the groups for each test (p>0.05; ANOVA and Tukey’s post-test for Knoop microhardness; Kruskal-Wallis for surface roughness).

### Knoop Microhardness

The incorporation of 2.5% and 5% β-AgVO_3_ resulted in a significant increase in Knoop microhardness values compared to the control group (p < 0.001) (Table 3).

### Roughness Test

**Fig 2:**
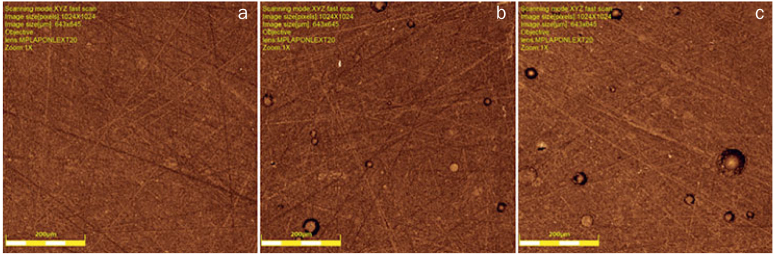
Confocal microscopy image of the roughness pattern. a: control group specimen; b: specimen with 2.5% β-AgVO_3_ incorporation; c: specimen with 5% β-AgVO_3_ incorporation.

Resin cement surface roughness increased significantly with the incorporation of the different concentrations (2.5% and 5%) of β-AgVO_3_ (p = 0.004). A significant difference was observed between the control and the 2.5% (p = 0.013) and 5% (p = 0.012) groups (Table 3) (Fig 2).

### Color Measurement

The data obtained for color evaluation are shown in Table 4.

**Table 4:** Values of mean and standard deviation (SD) for L*, a* and b* measured for each group and values of ΔE* calculated

Groups	CIELab System				
	L	a	b	ΔE 2.5%	ΔE 5%
Control	75.4 (1.6)A	0.2 (0.5)A	22.6 (2.0)A	19.7 (2.4)A	19.9 (4.6)A
2.5%	70.9 (0.7)B	6.3 (0.3) B	40.7 (1.2)B		
5%	71.8 (1.2)B	5.6 (0.5)C	41.3 (4.5)B		

Values of L, a, b, and ΔE followed by the same uppercase letters denote no statistically significant difference (p > 0.05).

**Fig 3:**
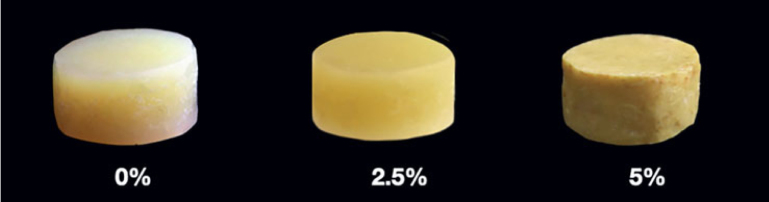
Cement specimens with β-AgVO_3._

The incorporation of β-AgVO_3_ at both concentrations yielded a significant difference in the color of the cement (Fig 3). For the L* and b* values, test groups showed statistically significant differences (p < 0.05) from the control group, but the 2.5% and 5% groups were similar to each other (p = 0.265). The a* value showed a significant difference between all groups (p < 0.05). Considering the color change (ΔE), the incorporation of β-AgVO_3_ showed no difference between the two concentrations tested.

### Shear Bond Strength

Before aging, different concentrations of β-AgVO_3_ (control, 2.5% and 5%) induced no statistically significant differences (p = 0.569) in SBS. The same was true after aging (p = 0.649).

However, when comparing the shear bond strengths before and after aging, the aged group 7.51 (3.27) MPa showed a significantly lower average (p = 0.046) (Table 5).

**Table 5:** Means and standard deviations (SD) of shear bond strength for different concentrations and times

	Before aging		After aging	p
Control	10.3 (4.6)	0.569	6.6 (2.1)	0.649
2.5%	8.7 (4.3)		7.5 (3.3)	
5%	11.2 (2.8)		8.5 (4.3)	
Total	10.1(3.9)		7.5 (3.3)	0.046

p > 0.05; ANOVA, Tukey’s post-hoc test.

### Analysis of Antimicrobial Activity

**Fig 4:**
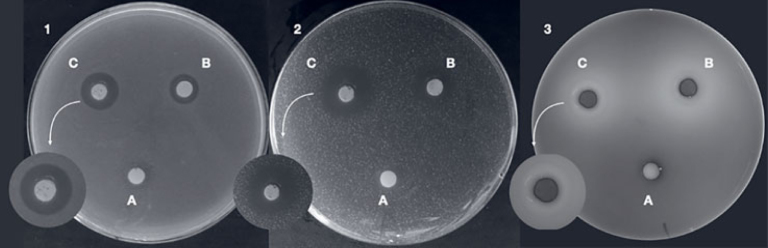
Agar diffusion test of cement specimens with β-AgVO_3_ incorporation. The letters show the concentrations. A: control group; B: 2.5% β-AgVO_3_ group; C: 5% β-AgVO_3_ group for microorganisms 1. *Staphylococcus aureus*, 2. *Streptococcus mutans*, 3. *Enterococcus faecalis.*

The control group, without the semiconductor, did not show any microbial inhibitory activity. All other groups exhibited antimicrobial activity against the selected species (p < 0.001). In general, increased inhibition occurred with increasing concentration of β-AgVO_3_, with the significantly highest antimicrobial activity for the 5% group (p < 0.001). When comparing the different microorganisms, the greatest antimicrobial activity was observed against *S. aureus* in the 2.5% and 5% β-AgVO_3_ groups (Table 6 and Fig 4).

**Table 6:** Inhibition halo (mm) of resin cement specimens incorporated with different percentages of nanostructured silver vanadate (β-AgVO_3_).

β-AgVO_3_	*S. aureus*	*S. mutans*	*E. faecallis*
	Mean (standard deviation)	Mean (standard deviation)	Mean (standard deviation)
2.5%	12.8 (0.8)Aa	8.3 (1.2)Ab	11.4 (0.6)Ac
5%	15.7 (1.0)Ba	14.9 (2.4)Bab	13.4 (0.9)Bb

Identical capital letters indicate statistical similarity between groups (β-AgVO_3_) for the same microorganism. The same lower-case letters indicate statistical similarity within the same group between different microorganisms (p>0.05; ANOVA and Tukey’s post-hoc test).

## Discussion

The present study evaluated the effect of incorporating the semiconductor β-AgVO_3_ into a dual-curing resin cement on the DC, microhardness, surface roughness, color, bond strength before and after artificial aging, and antimicrobial activity. Hypothesis 1 was partially accepted because the incorporation of β-AgVO_3_ in the resin cement maintained the DC and bond strength. Reductions in bond strength were considered to be an effect of thermal aging. However, there were statistically significant differences in surface microhardness, surface roughness, and color. Hypothesis 2 was accepted because the incorporation of the semiconductor provided antimicrobial efficacy against the tested microorganisms.

The DC of composites is defined as the percentage of double bonds between carbons (C=C) that are converted into single bonds during the polymerization process.^39^ The degree of conversion is never complete (ie, 100%).^8,37^ For bis-GMA–based resin composites the values vary from 52% to 75%,^4^ and depend on factors such as resin composition, filler particle size and loading, filler surface treatment, polymerization temperature (the higher the temperature, the higher the degree of conversion),^8^ and light absorption by the photoinitiator in the case of light-cured composites.^4,47^ A low DC with values less than 50% are inadvisable,^4,8^ as it reduces mechanical properties, changes dimensional stability, decreases adhesion to the dental structure, and results in microleakage, discoloration, solubility, degradation at the interface, and postoperative dental hypersensitivity.^18^

In this study, the conversion rates decreased after 48 h for the control sample. It is suggested that this occurred due to the lack of close contact between the sample and the ATR sensor of the FTIR spectrometer, as the same sample was repositioned on the ATR sensor for a new reading. ATR-FTIR spectroscopy is a versatile, non-destructive technique that makes it possible to quantify polymerization rates. It provides information about the curing time and identifies different chemical groups. It is also helpful for investigating the curing depth as the sample thickness increases. It also has the advantage that the spectrum can be read immediately after the cement has been mixed. However, it is crucial to ensure the best possible optical contact between the sample and the sensor. This condition is easier to fulfill when the film is built directly on the sensor. For solid samples, this contact limitation can be alleviated by applying a solid coating that causes the sample’s pressure. The most common method to improve the contact is using a mechanical clamp that presses the sample onto the ATR element. Another possibility is using a matrix to allow better optical contact between the solid sample and the sensor.^19,35,50^ In the present study, the sample was pressed through solid coverage. The observed decrease in the conversion rate was minimal for the groups with β-AgVO_3_, attesting to the stability between 10 min and 48 h. Thus, the dual-curing resin cement was not affected by the incorporation of β-AgVO_3_, corroborating the results presented by Dias et al,^20^ who observed no changes when silver nanoparticles were incorporated into a composite. Ahn et al^2^ and Poosti et al^49^ did not observe changes in the bond strength between composite and bracket, which may indicate that nanoparticles of Ag and TiO_2_ incorporated into an adhesive and an orthodontic composite did not interfere in the degree of conversion. In contrast, other authors^8^ observed that the higher the concentration of silver nanoparticles in composites containing bis-GMA/TEG-DMA was, the lower the degree of conversion, and this was probably caused by the increasing distance between the double bonds. In the present study, it can be suggested that there was no change in the DC, because the β-AgVO_3_ nanoparticles do not promote agglomeration. Thus, they can maintain adequate distance between the carbon double bonds.

When incorporated into the semiconductor matrix, metal nanoparticles result in antibacterial properties due to the catalytic effect.^12,17,63^ Reactive oxygen species (ROS) are generated through photocatalysis reactions when a metal oxide is exposed to light and generates free radicals, usually associated with oxygen-containing species. ROS can, due to their high reactivity, promote both antimicrobial activity and additional radical curing of not fully cross-linked resins. Thus, ROS do not negatively affect the resin polymerization; on the contrary – they can promote additional crosslinking and improve the resin’s performance.^56^

The DC and hardness are related; that is, if the cement is not sufficiently polymerized, both will be lower.^18,38^ However, in the present study, microhardness increased in groups with 2.5% and 5% β-AgVO_3_. These results are in accordance with those of Barszczewska-Rybarek and Chladek,^8^ who also found increased hardness values with the incorporation of 7% AgNPs in light-curing resin composites. Changes in hardness may be related to the amount, size, and shape of the reinforcement.^5^ Nanoparticles can promote additional reinforcement in the polymer cross-linked network.^11^ The smaller the particle size, the closer the contact it has with the resin matrix.^5^ Thus, this additional reinforcement is provided by covalent attachment between the surface of the nanoparticles and the resin matrix. Accordingly, it is suggested that the cement hardness increase is caused by the incorporation of semiconductor nanoparticles.^11^

Reports show that roughness is a favorable factor for microbial adhesion, as it provides retentive characteristics for bacteria.^5,24^ In this sense, the present study showed an increase in roughness for the groups with 2.5% and 5% β-AgVO_3_ (p = 0.004). These findings corroborate with other studies that also found increased values for roughness with the incorporation of Ag nanoparticles.^2,20^ However, Ahn et al^2^ found that composites containing silver nanoparticles prevented the adhesion of cariogenic streptococci, even though they had higher roughness values.^2^ Thus it is suggested that β-AgVO_3_ nanoparticles can overcome the presence of increased roughness and provide effective antibacterial activity.

A possible disadvantage of using silver in dentistry is the color.^2,36^ Values of ΔE ≥ 3.3 are clinically unacceptable, as they are noticeable to the untrained eye. In this study, the incorporation of the semiconductor showed noticeable differences. According to Štruncová et al,^55^ the color of the semiconductor conforms to its characteristics, as rounded corners or spherically shaped nanoparticles have a light yellow-orange color. The incorporation of silver nanoparticles can cause color changes due to the shadowing that such nanoparticles can cause.^26^ Another explanation for the color change is that as the concentration of silver nanoparticles increases, the color of the resins becomes darker due to the plasmonic effect of the AgNPs.^9,22^ This effect is similar to bending light around an object so that it is hidden. In addition, the interface between the fillers and the organic matrix can undergo hydrolysis and the silane can degrade. Temperature cycling can cause internal stresses and microcracks in the material as a result of linear thermal expansion coefficients. Thus, the color stability of a material depends on its composition and how the monomers and fillers react to chemical, physical, and thermal changes.^55^ Although a significant change in the color of the cement was observed, this situation would not inhibit the possible restorative/prosthetic use of this material, because most crowns, onlays, inlays, and bridges are not highly influenced by the background or the color of the cement.

Regarding adhesion, in this study, reduced bond strength was observed after aging, with values ranging from 6.60 MPa to 8.45 MPa. Nonetheless, these strengths are high enough to provide adequate adhesion of the bracket to the enamel and facilitate removal after the completion of orthodontic treatment, because they are greater than those suggested by Reynolds (5.9 to 7.8 MPa).^52^ Numerous other studies have also noted no significant difference in the bond strength of many resin materials (cements,^3,43,61^ adhesives,^42,62^ GIC resin-modified,^64,65^ and flowable resin^31^) when different substances were added to them (QAMs,^43^ silver/HA nanoparticles,^3,65^ calcium fluoride,^64^ and other semiconductors^61^). In this study, the addition of nanoAg showed no influence on the bond strength of Allcem. However, thermocycling is expected to reduce bond strength, which was the case in this study (p = 0.046). There are many different semiconductors, eg, TiO_2_, ZnO, CuO, SnO_2_, MgO, etc,^21,51,58^ some of which have been incorporated into dental materials, even showing the ability to improve adhesion.^31,61^ However, it was also observed that the bond strength decreased with high loads of ZnO-NPs. This might have been due to the agglomeration phenomenon, incomplete curing, or a composite with increased opacity.^7,61^

Nanotechnology has been applied in dentistry as an innovative concept to develop dental materials with better properties and anti-caries potential. The antimicrobial effectiveness of β-AgVO_3_ semiconductor has already been proven^13,33^ and has been evaluated by our research group for different dental materials. Kreve et al^39^ investigated the effect of β-AgVO_3_ incorporated into a reliner, which promoted antimicrobial activity without affecting the studied mechanical properties. Castro et al^13^ found that β-AgVO_3_, when incorporated into acrylic resin, inhibited the growth of *Candida albicans, Streptococcus mutans, Staphylococcus aureus,* and *Pseudomonas aeruginosa*. A 2.5% concentration of β-AgVO_3_ incorporated into irreversible hydrocolloid acted as an antimicrobial agent without affecting the physicomechanical properties.^14^

Using a semiconductor decorated with nano-silver has certain advantages compared to incorporating only nanosilver, as the use of AgNPs as antibacterial agents is generally limited. AgNPs tend to form agglomerates, and to prevent this from occurring, stabilizers are generally used, which decrease the antimicrobial efficacy. The semiconductor used in this study allows the AgNPs to be anchored to the silver vanadate nanowires, eliminating the need for stabilizers and preventing the formation of nanoparticle clumps. By keeping the nanoparticles dispersed, it is possible to maintain a high surface area of silver available for contact with bacteria.^32,33^

Even in patients who maintain good oral hygiene, orthodontic accessories hinder the mechanical removal of biofilm, increasing the predisposition for caries around orthodontic brackets and bands. In this context, the incorporation of β-AgVO_3_ semiconductor into the resin cement is beneficial, because bacterial activity can be achieved while maintaining acceptable bonding.

In terms of limitations, this study was not designed to answer all questions regarding the incorporation of the semiconductor β-AgVO_3_. Thus, further studies should investigate the effect of β-AgVO_3_ release from resin cement, as well as cytotoxic effects on fibroblasts, in order to enable the indication of this material without risks to the health of patients.

## Conclusion

It is suggested to incorporate the semiconductor β-AgVO_3_ at both concentrations into dual-curing resin cement. Moreover, the physicomechanical properties remained satisfactory for the proposed application.

## Clinical relevance:

The incorporation of the semiconductor into resin cement interferes with biofilm formation and maintains the mechanical properties of this material to a satisfactory degree.
